# Evaluation of the Vibrant DNA microarray for the high-throughput multiplex detection of enteric pathogens in clinical samples

**DOI:** 10.1186/s13099-019-0329-2

**Published:** 2019-10-18

**Authors:** Yuanyuan Yang, Vinod Rajendran, Vasanth Jayaraman, Tianhao Wang, Kang Bei, Karthik Krishna, Karenah Rajasekaran, John J. Rajasekaran, Hari Krishnamurthy

**Affiliations:** 1 Vibrant America LLC, San Carlos, CA USA; 2 Vibrant Sciences LLC, San Carlos, CA USA

**Keywords:** Multiplexing, Gastrointestinal infection, PCR, DNA microarray, Diarrhea

## Abstract

**Background:**

Rapid detection of a wide range of etiologic agents is essential for appropriate treatment and control of gastrointestinal (GI) infections. A variety of microbial species including bacteria, viruses, parasites, and fungi have been recognized as diarrheagenic enteric pathogens. However, multiplex testing of various targets in a single reaction needs further improvement because of its limitation in species and throughput.

**Results:**

This study aims at developing and evaluating a DNA microarray-based qualitative multiplexed polymerase chain reaction (PCR) assay, Vibrant GI pathogen panel (GPP), for simultaneous detection of 27 enteric GI pathogenic targets (16 bacteria, 5 viruses, 4 parasites, and 2 fungi) directly from stool specimens. Limits of detection ranged from 10^2^ to 10^4^ cells/mL for bacteria, 10^2^ to 10^3^ cells/mL for parasites, 10^2^ to 10^3^ RNA copies/mL for viruses, and 10^2^ to 10^3^ cells/mL for fungi. Performance characteristics were determined using 27 Quantitative Genomic DNAs, 212 spiked stool specimens, 1067 clinical and archived stool specimens. Overall sensitivity was 95.9% (95% CI 92.4–98.1) and specificity was 100% (95% CI 99.9–100). Polymicrobial detections contained either two or three organisms was 20.2% (35/173) of positive clinical specimens and 3.3% (35/1055) of all clinical specimens.

**Conclusion:**

The Vibrant GPP is a comprehensive, high-throughput, and rapid DNA microarray to provide etiologic diagnosis of GI infections in the laboratory setting.

## Introduction

Infectious diarrhea is a leading cause of global morbidity and mortality, which contributes to the death of around one million children globally each year [1, 2]. A variety of bacteria, viruses, and parasites can cause gastrointestinal (GI) infections that manifest as inflammation of the stomach and intestines [3, 4]. A healthcare practitioner may suspect the infectious agents based upon a person’s recent food and drink, medical history, and/or recent travel but will not be able to positively identify the pathogen without laboratory testing [5]. Different diagnostic modalities are available to provide qualitative and/or quantitative results but all have inherent limitations. Culture methods are relatively low yield and less accurate for enteric pathogens, especially unfavorable to be used in antibiotic treated samples [6]. Microscopy is usually used for parasite detection due to its low cost but also involves requirement of highly-skilled parasitologist and longer turnaround time [7]. Antigen-based tests provide advanced diagnostic results for diarrheal; however, not all relevant pathogens have been determined with this method [8]. Molecular tests, as we presented in this study, have the potential to overcome the above issues and provide new opportunities to detect enteric pathogens.

Rapid and accurate determination of GI pathogens in severe cases is vitally important to aid decision making so that appropriate treatment, isolation, management, and further investigations can be initiated [9]. A GI pathogen panel (GPP), which exploit multiplex nucleic acid amplification methodology, can detect the genetic materials (RNA or DNA) of a wide range of the more common microbes and identify the presence of pathogenic microbes and co-infections from human stool specimens in a single run [10]. A GPP test can potentially increase the throughput and volume of information and decrease the turnaround-time [11]. Moreover, the ability to rapidly and accurately identify the pathogens in GI infected samples has become particularly important to aid in the diagnosis of GI infections, tracing of contact, and management of diseases. However, most currently available multiplex GPPs have their own limitations, such as insufficient clinical sensitivity and difficulty in incorporating additional assays when new species or subtypes emerge.

In this study, we developed and evaluated the Vibrant GPP, which is a DNA microarray-based qualitative multiplexed polymerase chain reaction (PCR) assay intended for use in simultaneous detection and identification of nucleic acids from multiple GI pathogens directly from the stool samples obtained from individuals with GI infection symptoms. The Vibrant GPP is a microarray-based panel containing 27 enteric GI pathogenic targets (16 bacteria, 5 viruses, 4 parasites, and 2 fungi). We examined the performance characteristics of this multiplex GPP and compared with the routine GI infection diagnostic assays in the laboratory setting.

## Materials and methods

### Quantitative genomic DNAs and stool specimens

27 Quantitative Genomic DNAs were obtained from American Type Culture Collection (ATCC) (Manassas, VA USA), ZeptoMetrix (Buffalo, NY), and Waterborne (New Orleans, LA) including *E. coli* O157:H7 (ATCC 43895), Enteroaggregative *E. coli* (ATCC 23501), Enteropathogenic *E. coli* (ATCC 43887), Enterotoxigenic *E. coli* (ETEC) lt/st (ATCC 35401), Shiga-like toxin producing *E. coli* (STEC) stx1/stx2 (ATCC BAA-2196, ATCC 43895), *Plesiomonas shigelloides* (ATCC 14029), *Vibrio parahaemolyticus* (ATCC 17802), *Vibrio vulnificus* (ATCC 27562), *Helicobacter pylori* (ATCC 700392), *Listeria* spp. (ATCC 19111), *Vibrio cholerae* (ATCC 14035), *C. difficile* Toxin A/B (ATCC 9689), *Salmonella* spp. (ATCC 700623), *Shigella*/Enteroinvasive *E. coli* (ATCC 29930), *Yersinia enterocolitica* (ATCC 9610), *Campylobacter jejuni* (ATCC BAA-1234), *Campylobacter upsaliensis* (ATCC 43954), *Giardia lamblia* (ATCC 30957), *Cryptosporodium* spp. (Waterborne P102C), *Entamoeba histolytica* (ATCC 30459), *Cyclospora cayetanensis* (Zeptometrix control), Norovirus GI/GII (Zeptometrix control), Rotavirus A (ATCC VR-2104), Adenovirus F 40/41 (ATTC VR-930/931), Astrovirus (ATCC VR-3238SD), Sapovirus (I, II, IV, V) (Zeptometrix control), *Candida* spp. (ATCC 10231). The isolates from ATCC were cultured on blood agar plates or desired media based on ATCC guidelines (https://www.atcc.org/Guides.aspx) and stored at − 80 °C in CryoBeads (Hardydiagnostics, Santa Maria, CA) along with a cryopreservative liquid (Brucella Broth with Glycerol). Parasitic, viral and fungal isolates were obtained as measured suspensions from ZeptoMetrix (#NATGIP-BIO) and were stored based on manufactures’ guidelines.

A total of 1067 clinical and archived stool specimens were collected between June 2015 to June 2017 and tested in the Vibrant America Clinical Laboratory. Stool specimens were transported in Cary-Blair transport media. The waiver of consent for In Vitro Diagnostic Device study using leftover human specimens that are not individually identifiable was approved by the Western Institutional Review Board (WIRB) (work order #1-1098539-1). The inclusion criteria for clinical stool specimens were: subjects’ Cary-Blair enteric transport medium containing sufficient volume for testing and could be tested via the VG-GPP within 4 days of specimen collection (stored at 4 °C). The exclusion criteria for the stool specimens were: subjects with complex GI disorders which may interfere with an accurate diagnostic decision.

### Vibrant gastrointestinal pathogens panel (GPP)

Vibrant Gastrointestinal Pathogens Panel (GPP) is a multiplexed qualitative test for simultaneous detection of nucleic acids from 27 different pathogens including bacterial, viral, parasitic, and fungal species (complete list seen in Table 1) in human stool specimens from individuals with signs and symptoms of GI infections. Genomic DNA and RNA were extracted using commercial extraction kit purchased from Omega Biotek (Norcross, GA). During the PCR process, sequence-specific primers directed the amplification of target DNA with amplicon size 200 bp. Followed by PCR, DNA sequences were hybridized to sequence-specific probes immobilized on the silicon chip surface and labelled by an on-chip enzyme-based labelling technique. The unbound conjugates were washed away. Luminol was added to produce a chemiluminescent signal at the location of the probe/target sequence complex. The resulting signal was detected by a charge coupled device (CCD) imaging system along with the Vibrant TSP Software (Vibrant Sciences LLC, San Carlos, CA) for array mapping and data analysis.

**Table 1:** GI pathogens detected by the Vibrant GPP

Bacteria	*Clostridium difficile* toxin A/B
	*Campylobacter* spp*. (jejuni, upsaliensis)*
	*Plesiomonas shigelloides*
	*Yersinia enterocolitica*
	*Salmonella* spp.
	*Vibrio parahaemolyticus*
	*Vibrio cholerae*
	*Vibrio vulnificus*
	Enteroaggregative *E. coli* (EAEC)
	Enteropathogenic *E. coli* (EPEC)
	Enterotoxigenic *E. coli* (ETEC) lt/st
	Shiga-like toxin producing *E. coli* (STEC) stx1/stx2
	*E. coli* O157:H7
	*Shigella*/Enteroinvasive *E. coli* (EIEC)
	*Helicobacter pylori*
	*Listeria* spp.
Virus	Norovirus GI/GII
	Rotavirus A
	Adenovirus F 40/41
	Astrovirus
	Sapovirus (I, II, IV, V)
Parasite	*Giardia lamblia*
	*Cryptosporidium* spp*. (parvum, hominis)*
	*Entamoeba histolytica*
	*Cyclospora cayetanensis*
Fungi	*Candida* spp.
	*Microsporidium* spp.

### Pathogen-specific primer design

The primer setup was designed to target the ribosomal RNA genes (16S or 23S) of the bacterial groups and accession numbers of the GenBank sequences that we used as reference for parasitic, viral and fungal organisms were MF962514.1, KM099402.1, MG571777.1, MH520738.1, MG692437.1, MG266048.1, KY658153.1, XR_003297358.1, LC341260.1, and CP025165.1. Specific primers were designed using the Primer-blast tool, and further validated based on BLAST search (https://blast.ncbi.nlm.nih.gov). Primers were designed to have approximately same lengths of nucleotides, GC-content, and to produce amplicons between 100 and 250 bp long. Some of the published primers were slightly modified to improve their specificity.

### Nucleic acid extraction

In this study, we used a commercial kit (Omega Biotek, Norcross, GA) for the extraction and purification of total pathogenic DNA/RNA from stool specimens. Prior to extraction, samples stored in the Para-Pak C&S transport media were thawed and centrifuged at 5000 rpm for 10 min. The samples were diluted with sterile phosphate buffered solution to remove excess debris from supernatant solution. Individual fecal aliquots were processed according to the manufacturer’s instructions specified in the kit with minor modifications. This procedure included lysis, protein degradation, and DNA/RNA purification. A portion of 250 µL from each fecal specimen was transferred into the bead’s container. Subsequently, portions of 500 µL SLX-MLUS buffer and 20 µL proteinase were added to the same container. The samples were mixed by vortexing and centrifuged at 3500 rpm for 2 min. The samples were homogenized by bead beating with Geno Grinder 2000 at 1000 stokes/min for 10 min and then centrifuged twice at 3500 rpm for 2 min. The sample was then heated at 70 °C for 10 min and subsequently centrifuged twice at 4500 rpm for 5 min. An aliquot of 500 µL clear supernatant was mixed with 600 µL of RBB Buffer, 300 µL XP2 Buffer, 20 µL of Omega Mag-Bind Beads by vortexing for 15 min. The mixture was placed on the magnetic station for 90 s and the supernatant was removed. The magnetic beads were washed with 750 µL VHB buffer and SPM buffer. Finally, DNA/RNA was eluted from the beads by incubation with 200 µL elution buffer. The concentration and quality of the extracted nucleic acids were measured spectrophotometrically using a NanoDrop™ ND-1000 spectrophotometer (NanoDrop Technologies Inc., Wilmington, DE). Positive and negative controls (Zeptometrix #NATGIP-BIO) were used in the DNA/RNA extraction procedure.

### Multiplex PCR amplification

GPP Multiplex PCR Master Mix (Vibrant Sciences LLC, San Carlos, CA) was developed for efficient simultaneous detection of GI pathogens. The GPP MUX Primer Mix contained 5.00 µM GPP *Campylobacter*.X8201, 2.50 µM GPP *Plesiomonas shigelloides*.X8202, 5.00 µM GPP *Yersinia enterocolitica*.X8203, 5.00 µM GPP *Salmonella*.X8204, 5.00 µM GPP *Vibrio parahaemolyticus*.X8205, 5.00 µM GPP *Vibrio cholerae*.X8206, 5.00 µM GPP *Vibrio vulnificus*.X8207, 1.25 µM GPP Enteroaggregative *E. coli* (EAEC).X8208, 1.25 µM GPP Enteropathogenic *E. coli* (EPEC).X8209, 1.25 µM GPP Enterotoxigenic *E. coli* (ETEC) lt/st.X8210, 5.00 µM GPP STEC stx1/stx2.X8211, 5.00 µM GPP *E. coli* O157.X8212, 1.25 µM GPP Enteroinvasive *E. coli* (EIEC).X8213, 5.00 µM GPP *Helicobacter pylori*.X8214, 5.00 µM GPP *Listeria* spp.X8215, 1.00 µM GPP Norovirus GI/GII.X8216, 1.00 µM GPP Rotavirus A.X8217, 1.00 µM GPP Adenovirus.X8218, 1.00 µM GPP Astrovirus.X8219, 1.00 µM GPP Sapovirus.X8220, 5.00 µM GPP *Giardia lamblia*.X8221, 5.00 µM GPP *Cryptosporidium*.X8222, 5.00 µM GPP *Entamoeba histolytica*.X8223, 5.00 µM GPP *Cyclospora cayetanensis*.X8224, 0.50 µM GPP *Candida* spp.X8226, 0.50 µM GPP *Microsporidium* spp.X8228. The GPP Multiplex PCR Master Mix was prepared and distributed into 50 µL aliquots. The mixture contained 25 µL PCR buffer which was prepared with 200 mM Tris–HCl, pH 8.4, 250 mM KCl, 2.50 mM MgCl_2_, 0.25 mM of each deoxynucleotide triphosphate (dATP, dCTP, dGTP. dTTP), 2.0 µL GPP MUX Primer MIX, 0.5 µL of 0.50 M Dimethyl sulfoxide (DMSO), 1.0 µL of Titanium Taq DNA Polymerase (TaKaRa Bio US, Inc., Mountain View, CA), and 20.5 µL DNase/RNase-Free Distilled Water (Thermofisher Scientific, Waltham, MA). A portion of 50 µL master mix was used in each PCR reaction. The final mixture was aliquoted into 96 well PCR Well plate along with 1.0 µL extracted nucleic acid. The amplification reactions were performed in a Mastercycler Pro (Eppendorf, Hauppauge, NY). First, an initial incubation at 95 °C for 10 min was performed, followed by 50 amplification cycles consisting of denaturation at 95 °C for 30 s, primer annealing at 60 °C for 30 s, and extension 72 °C for 1 min. The final extension was at 72 °C for 5 min. Positive and negative controls (Zeptometrix #NATGIP-BIO) were used in the multiplex PCR amplification procedure.

### GPP array hybridization

The Vibrant GPP Arrays (Vibrant Sciences LLC, San Carlos, CA) were pre-blocked with 150 µL GPP Blocking Buffer in a hybridization oven for 30 min at 37 °C. After 30-minute blocking, the solution was discarded and 300 µL GPP Wash Buffer was dispensed into each well of a 24-well plate (Costar, Corning, NY). The array was put back and the plate was vortexed for 2 min at 350 rpm. Following each step, each array was washed thrice with 300 µL GPP Wash Buffer to remove any nonspecific binding. The PCR product containing 50 µL target DNA was added to a 24 well plate and mixed by pipette along with 20 µL GPP Denaturing Buffer. The plate was then sealed and vortexed for 10 min at room temperature at 650 rpm. Then 100 µL GPP Prehybridization Buffer was dispensed in each well of a 24 well plate (Costar, Corning, NY) before being placed with the array. After a 2-h hybridization at 55 °C, the solution was discarded and 300 µL GPP Wash Buffer was dispensed in each well of a 24 well plate. The array was again put back and the plate was vortexed for 2 min at 350 rpm.

### GPP array on-chip extension and labelling

For on-chip extension and labelling, the GPP Extension Master Mix was prepared by adding 100 µL GPP Extension Mix consisted of 100 mM pH 8.4 Tris–HCl, 150 mM KCl, 0.5 mM MgCl_2_, 0.25 mM of each deoxynucleotide triphosphate (dATP, dGTP, dTTP), 0.1 µmol of dCTP, 1 mM final concentration of Biotin-16-dCTP, and 2.5 µL DNA Polymerase I. Once the enzyme was added to the GPP Extension Master Mix, the whole mixture was applied to the array. The reaction was allowed for 30 min at 55 °C in a hybridization oven. The solution was discarded and 300 µL GPP Wash Buffer was dispensed in each well of a 24 well plate. The array was put back and the plate was vortexed for 2 min at 350 rpm. The resulting biotin-labeled DNA probes were subsequently detected using streptavidin conjugated with horseradish peroxidase (HRP) system. For each reaction, 250 µL GPP Detection Mix was added to each well of a 24 well plate and the array was incubated for 15 min at room temperature. The array was then washed thrice with 300 µL GPP Wash Buffer to remove non-conjugated probes. Positive and negative controls (Zeptometrix #NATGIP-BIO) were used in the on-chip extension procedure.

### GPP array target detection

The HRP-tagged Arrays were placed in the CCD imaging system along with 250 µL luminol-based detection substrates. The reactions were read by the instrument and median chemiluminescence intensities were exported to the Vibrant TSP Software (Vibrant Sciences LLC, San Carlos, CA) for array mapping and data analysis.

## Results

### Precision study

A total of 27 Quantitative Genomic DNAs of the pathogenic targets were tested by the Vibrant GPP. Each organism was tested repeatedly for 20 times (2 operators, 2 runs per operator, 5 repeats per run). The assay was able to detect all of these organisms and responded at the exact concentration level, as shown in Table 2.

**Table 2:** The Vibrant GPP array's performance evaluation with the quantitative genomic DNAs

Organism Source/isolate ID	Target concentration	Agreement
*E. coli* O157:H7	6.0 × 10^4^ cells/mL	100% (20/20)
ATCC 43895		
Enteroaggregative *E. coli* (EAEC)	4.8 × 10^6^ cells/mL	100% (20/20)
ATCC 23501		
Enteropathogenic *E. coli* (EPEC)	3.6 × 10^5^ cells/mL	100% (20/20)
ATCC 43887		
Enterotoxigenic *E. coli* (ETEC) lt/st	3.6 × 10^7^ cells/mL	100% (20/20)
ATCC 35401		
Shiga-like toxin producing *E. coli* (STEC) stx1/stx2	2.8 × 10^5^ cells/mL	100% (20/20)
ATCC BAA-2196, ATCC 43895		
*Plesiomonas shigelloides*	6.0 × 10^5^ cells/mL	100% (20/20)
ATCC 14029		
*Vibrio parahaemolyticus*	6.0 × 10^5^ cells/mL	100% (20/20)
ATCC 17802		
*Vibrio vulnificus*	2.4 × 10^8^ cells/mL	100% (20/20)
ATCC 27562		
*Helicobacter pylori*	3.6 × 10^8^ cells/mL	100% (20/20)
ATCC 700392		
*Listeria* spp.	6.0 × 10^6^ cells/mL	100% (20/20)
ATCC 19111		
*Vibrio cholerae*	6.0 × 10^5^ cells/mL	100% (20/20)
ATCC 14035		
*C. difficile* Toxin A/B	3.6 × 10^5^ cells/mL	100% (20/20)
ATCC 9689, Clinical Specimen		
*Salmonella* spp.	4.8 × 10^6^ cells/mL	100% (20/20)
ATCC 700623		
*Shigella*/Enteroinvasive *E. coli* (EIEC)	2.4 × 10^5^ cells/mL	100% (20/20)
ATCC 29930		
*Yersinia enterocolitica*	4.8 × 10^8^ cells/mL	100% (20/20)
ATCC 9610		
*Campylobacter jejuni*	4.8 × 10^6^ cells/mL	100% (20/20)
ATCC BAA-1234		
*Campylobacter upsaliensis*	4.8 × 10^3^ cells/mL	100% (20/20)
ATCC 43954		
*Giardia lamblia*	3.6 × 10^4^ cells/mL	100% (20/20)
ATCC 30957		
*Cryptosporodium* spp.	1.5 × 10^4^ oocycts/mL	100% (20/20)
Waterborne P102C		
*Entamoeba histolytica*	2.4 × 10^3^ cells/mL	100% (20/20)
ATCC 30459		
*Cyclospora cayetanensis*	2.4 × 10^5^ RNA copies/mL	100% (20/20)
Zeptometrix control		
Norovirus GI/GII	1.1 × 10^5^ RNA copies/mL	100% (20/20)
Zeptometrix control, Clinical Specimen		
Rotavirus A	1.0 TCID50/mL	100% (20/20)
ATCC VR-2104		
Adenovirus F 40/41	1.0 TCID50/mL	100% (20/20)
ATTC VR-930/931		
Astrovirus	1.2 × 10^3^ RNA copies/mL	100% (20/20)
ATCC VR-3238SD		
Sapovirus (I, II, IV, V)	2.1 × 10^5^ RNA copies/mL	100% (20/20)
Zeptometrix control		
Candida spp.	2.4 × 10^3^ cells/mL	100% (20/20)
ATCC 10231		
*Microsporidium* spp.	2.2 × 10^5^ DNA copies/mL	100% (20/20)
Clinical Specimen		

### Limit of detection analysis

Limit of detection (LoD) for each pathogenic species was determined at the lowest concentration that the organisms can be consistently detected (≥ 95% of samples test positive). The LoD for each species was estimated with limiting dilutions in single-spiked samples. The LoDs were determined by testing a series of 1:5 dilutions of organism-spiked stool samples at known cell concentrations (e.g., 1 × 10^6^ cells/mL) and genomic DNA/cDNA concentrations (ranging from 1 × 10^−3^ to 2 µg/mL). Confirmation of LoDs was performed by spiking the target species at the LoD estimates determined by the dilution test and obtained from at least 5 of the 5 samples. Overall observations from the analysis indicate that the bacteria’s LoD range from 10^2^ to 10^4^ cells/mL; parasites’ LoD was 10^2^ to 10^3^ cells/mL; viruses’ LoD was 10^2^ to 10^3^ RNA copies/mL, fungi’s LoD was 10^2^ to 10^3^ cells/mL. The LoDs of each pathogenic target on the Vibrant GPP are presented in Table 3.

**Table 3:** The Vibrant GPP array’s lowest limit of detection

	Organism	Source/isolate ID	LoD concentration	Agreement
Bacteria	*E. coli* O157:H7	ATCC 43895	1.0 × 10^2^ cells/mL	100% (5/5)
	Enteroaggregative *E. coli* (EAEC)	ATCC 23501	1.0 × 10^2^ cells/mL	100% (5/5)
	Enteropathogenic *E. coli* (EPEC)	ATCC 43887, Clinical Specimen	1.5 × 10^3^ cells/mL	100% (5/5)
	Enterotoxigenic *E. coli* (ETEC) lt/st	ATCC 35401	2.0 × 10^3^ cells/mL	100% (5/5)
	Shiga-like toxin producing *E. coli* (STEC) stx1/stx2	ATCC BAA-2196, ATCC 43895, Clinical Specimen	1.0 × 10^2^ cells/mL	100% (5/5)
	*Plesiomonas shigelloides*	ATCC 14029	3.0 × 10^2^ cells/mL	100% (5/5)
	*Vibrio parahaemolyticus*	ATCC 17802	3.0 × 10^3^ cells/mL	100% (5/5)
	*Vibrio vulnificus*	ATCC 27562	1.0 × 10^4^ cells/mL	100% (5/5)
	*Helicobacter pylori*	ATCC 700392	1.5 × 10^4^ cells/mL	100% (5/5)
	*Listeria* spp.	ATCC 19111	3.0 × 10^3^ cells/mL	100% (5/5)
	*Vibrio Cholerae*	ATCC 14035	2.0 × 10^2^ cells/mL	100% (5/5)
	*C. difficile* Toxin A/B	ATCC 9689	1.0 × 10^3^ cells/mL	100% (5/5)
	*Salmonella* spp.	ATCC 700623	2.0 × 10^3^ cells/mL	100% (5/5)
	*Shigella/*Enteroinvasive *E. coli* (EIEC)	ATCC 29930	1.0 × 10^2^ cells/mL	100% (5/5)
	*Yersinia enterocolitica*	ATCC 9610	2.0 × 10^4^ cells/mL	100% (5/5)
	*Campylobacter jejuni*	ATCC BAA-1234	3.0 × 10^2^ cells/mL	100% (5/5)
	*Campylobacter upsaliensis*	ATCC 43954	1.0 × 10^2^ cells/mL	100% (5/5)
Parasite	*Giardia lamblia*	ATCC 30957	4.0 × 10^2^ cells/mL	100% (5/5)
	*Cryptosporodium* spp.	Waterborne P102C	1.0 × 10^2^ oocycts/mL	100% (5/5)
	*Entamoeba histolytica*	ATCC 30459	1.0 × 10^2^ cells/mL	100% (5/5)
	*Cyclospora cayetanensis*	Zeptometrix control	2.0 × 10^3^ DNA copies/mL	100% (5/5)
Virus	Norovirus GI/GII	Zeptometrix control	1.0 × 10^3^ RNA copies/mL	100% (5/5)
	Rotavirus A	ATCC VR-2104	3.1 × 10^2^ RNA copies/mL	100% (5/5)
	Adenovirus F 40/41	ATTC VR-930/931	1.0 × 10^2^ RNA copies/mL	100% (5/5)
	Astrovirus	Zeptometrix control	1.2 × 10^3^ RNA copies/mL	100% (5/5)
	Sapovirus (I, II, IV, V)	Zeptometrix control	2.1 × 10^2^ RNA copies/mL	100% (5/5)
Fungi	*Candida* spp.	ATCC 10231	1.0 × 10^2^ cells/mL	100% (5/5)
	*Microsporidium* spp.	Clinical specimen	2.0 × 10^2^ DNA copies/mL	100% (5/5)

### Performance evaluation of validated positive specimens

A total of 51 culture isolates spiked to negative stool specimens along with 161 clinical positive stool specimens collected by the Vibrant America Biorepository were tested with the Vibrant GPP. This DNA microarray was able to confirm all of the previously identified pathogens with 100% correlation when compared with the culture and RT-PCR (operation procedures in Additional files 1, 2), as shown in Table 4.

**Table 4:** The Vibrant GPP array's performance evaluation with validated positive specimens

Organism	Comparator (culture or RT-PCR)		Vibrant GPP array		Agreement (95% CI)
	Culture isolates	Clinical samples	Culture isolates	Clinical samples	
Bacteria					
*E. coli* O157:H7	2	7	2	7	100 (66.4–100)
Enteroaggregative *E. coli* (EAEC)	2	3	2	3	100 (47.8–100)
Enteropathogenic *E. coli* (EPEC)	1	4	1	4	100 (47.8–100)
Enterotoxigenic *E. coli* (ETEC) lt/st	2	6	2	6	100 (63.1–100)
Shiga-like toxin producing *E. coli* (STEC) stx1/stx2	2	9	2	9	100 (71.5–100)
*Plesiomonas shigelloides*	1	8	1	8	100 (66.4–100)
*Vibrio parahaemolyticus*	1	7	1	7	100 (63.1–100)
*Vibrio vulnificus*	1	6	1	6	100 (59.0–100)
*Helicobacter pylori*	2	3	2	3	100 (47.8–100)
*Listeria* spp.	1	6	1	6	100 (59.0–100)
*Vibrio cholerae*	1	3	1	3	100 (39.8–100)
*C. difficile* toxin A/B	1	9	1	9	100 (69.2–100)
*Salmonella* spp.	3	8	3	8	100 (71.5–100)
*Shigella/*Enteroinvasive *E. coli* (EIEC)	3	5	3	5	100 (63.1–100)
*Yersinia enterocolitica*	1	5	1	5	100 (54.1–100)
*Campylobacter jejuni*	2	3	2	3	100 (47.8–100)
*Campylobacter upsaliensis*	1	3	1	3	100 (39.8–100)
Parasite					
*Giardia lamblia*	1	3	1	3	100% (39.8–100)
*Cryptosporodium* spp.	2	8	2	8	100% (69.2–100)
*Entamoeba histolytica*	7	2	7	2	100% (66.4–100)
*Cyclospora cayetanensis*	2	4	2	4	100% (54.1–100)
Virus					
Norovirus GI	2	9	2	9	100% (71.5–100)
Norovirus GII	2	3	2	3	100% (47.8–100)
Rotavirus A	1	7	1	7	100% (63.1–100)
Adenovirus F 40/41	1	10	1	10	100% (71.5–100)
Astrovirus	1	9	1	9	100% (69.2–100)
Sapovirus (I, II, IV, V)	1	2	1	2	100% (29.2–100)
Fungi					
*Candida* spp.	1	6	1	6	100% (59.0–100)
*Microsporidium* spp.	1	1	1	1	100% (15.8–100)

**P < 0.00001 (Chi square test) for culture versus the Vibrant GPP array

### Accuracy study

A total of 1055 prospective clinical stool specimens were tested by the Vibrant GPP and the results were compared with conventional culturing methods and RT-PCR (operation procedures are detailed in Additional files 1, 2). As shown in Table 5, the three organisms that were the most prevalent in this cohort were: *E. coli* O157:H7, EPEC, and *Candida* spp. Overall sensitivity was 95.9% (95% CI 92.4–98.1) and specificity was 100% (95% CI 99.9–100). Individual targets’ sensitivity, specificity, positive predictive value (PPV), negative predictive value (NPV) are reported in Table 5 along with their 95% CI ranges.

**Table 5:** Clinical performance of the Vibrant GPP array with clinical samples

Vibrant GPP panel	No. of positive samples	No. of negative samples	Sensitivity % (95% CI)	Specificity % (95% CI)	PPV % (95% CI)	NPV % (95% CI)
*E. coli* O157:H7	25	1028	96.2 (80.4–99.9)	99.9 (99.5–100)	96.2 (80.4–99.9)	99.9 (99.5–100)
Enteroaggregative *E. coli* (EAEC)	8	1046	100 (63.1–100)	99.9 (99.5–100)	88.9 (63.1–100)	100 (99.5–100)
Enteropathogenic *E. coli* (EPEC)	17	1038	100 (80.5–100)	100 (99.6–100)	100 (80.5–100)	100 (99.6–100)
Enterotoxigenic *E. coli* (ETEC) lt/st	6	1047	100 (54.1–100)	99.8 (99.3–100)	75.0 (54.1–100)	100 (99.3–100)
Shiga-like toxin producing *E. coli* (STEC) stx1/stx2	12	1042	92.3 (64.0–99.8)	100 (99.6–100)	100 (64.0–99.8)	99.9 (99.6–100)
*Plesiomonas shigelloides*	4	1050	80 (28.4–99.5)	100 (99.6–100)	100 (28.4–99.5)	99 (99.6–100)
*Helicobacter pylori*	4	1051	100 (39.8–100)	100 (99.6–100)	100 (39.8–100)	100 (99.6–100)
*Listeria* spp.	7	1048	100 (59.0–100)	100 (99.6–100)	100 (59.0–100)	100 (99.6–100)
*Vibrio cholerae*	5	1048	100 (47.8–100)	99.8 (99.3–100)	71.4 (47.8–100)	100 (99.3–100)
*C. difficile* Toxin A/B	10	1043	91.7 (61.5–99.8)	99.9 (99.5–100)	91.7 (61.5–99.8)	99.9 (99.5–100)
*Salmonella* spp.	13	1042	100 (75.3–100)	100 (99.6–100)	100 (75.3–100)	100 (99.6–100)
*Shigella*/Enteroinvasive *E. coli* (EIEC)	5	1049	83.3 (35.9–99.6)	100 (99.6–100)	100 (35.9–99.6)	99.9 (99.6–100)
*Yersinia enterocolitica*	12	1042	92.3 (64.0–99.8)	100 (99.6–100)	100 (64.0–99.8)	99.9 (99.6–100)
*Campylobacter jejuni*	7	1047	87.5 (47.4–99.7)	100 (99.6–100)	100 (47.4–99.7)	99.9 (99.6–100)
*Campylobacter upsaliensis*	3	1051	100 (30.0–100)	99.9 (99.5–100)	75.0 (30.0–100)	100 (99.5–100)
*Giardia lamblia*	8	1047	100 (63.1–100)	100 (99.6–100)	100 (63.1–100)	100 (99.6–100)
*Entamoeba histolytica*	6	1049	100 (54.1–100)	100 (99.6–100)	100 (54.1–100)	100 (99.6–100)
*Cyclospora cayetanensis*	3	1050	100 (30.0–100)	99.8 (99.3–100)	60.0 (30.0–100)	100 (99.3–100)
Norovirus GI/GII	12	1042	92.3 (64.0–99.8)	100 (99.6–100)	100 (64.0–99.8)	99.9 (99.6–100)
Rotavirus A	4	1051	100 (39.8–100)	100 (99.6–100)	100 (39.8–100)	100 (99.6–100)
Adenovirus F 40/41	7	1048	100 (59.0–100)	100 (99.6–100)	100 (59.0–100)	100 (99.6–100)
Astrovirus	4	1052	100 (39.8–100)	100 (99.6–100)	100 (39.8–100)	100 (99.6–100)
Sapovirus (I, II, IV, V)	3	1050	100 (47.8–100)	100 (99.6–100)	100 (47.8–100)	100% (99.6–100)
*Candida* spp.	26	1027	96.3 (81.0–99.9)	99.8 (99.3–100)	92.9 (81.0–99.9)	99.9 (99.3–100)

Several pathogenetic targets were not encountered in this cohort. To supplement the results of the prospective clinical study, 12 archived clinical samples were added to the original pool. These specimens were organized into the testing pool and randomized such that the users performing the Vibrant GPP were blinded as to the expected test result. A summary of the testing results for these archived samples are presented in Table 6. Overall sensitivity for these archived clinical samples was 100% (95% CI 73.5–100) and specificity was 100% (95% CI 99.9–100). Individual target’s sensitivity, specificity, positive predictive value (PPV), negative predictive value (NPV) are reported in Table 6 along with their 95% CI ranges.

**Table 6:** Clinical performance of the Vibrant GPP array with archived clinical specimens

Vibrant GPP panel	No: positive samples tested	No: negative samples tested	Sensitivity % (95% CI)	Specificity % (95% CI)	PPV % (95% CI)	NPV % (95% CI)
*Vibrio parahaemolyticus*	3	1055	100 (29.2–100)	100 (99.6–100)	100 (29.2–100)	100 (99.6–100)
*Vibrio vulnificus*	3	1055	100 (29.2–100)	100 (99.6–100)	100 (29.2–100)	100 (99.6–100)
*Cryptosporodium* spp.	1	1055	100 (2.5–100)	100 (99.6–100)	100 (2.5–100)	100 (99.6–100)
*Microsporidium* spp.	2	1055	100 (15.8–100)	100 (99.6–100)	100 (15.8–100)	100 (99.6–100)

### Detection of multiple pathogens

Among the 1055 clinical specimens, the Vibrant GPP reported polymicrobial detections (i.e., mixed infections) for a total 35 specimens, as shown in Table 7. This represents 20.2% (35/173) of positive samples and 3.3% (35/1055) of all samples. The multiple detections contained either two or three organisms. The three organisms that were the most prevalent in co-infections were: *E. coli* O157:H7, STEC stx1/stx2, EPEC. All of the samples with multiple pathogens were concordant with the reference methods.

**Table 7:** Most prevalent co-infections detected by the Vibrant GPP array

Multiple detection	Number of specimens
*E. coli* O157:H7 + Norovirus (GI/GII)	10
*Campylobacter jejuni* + *E. coli* O157:H7 + STEC stx1/stx2	1
*C. difficile* toxin A/B + STEC stx1/stx2	3
Adenovirus 40/41 + EPEC	5
*Candida* spp. + EPEC	3
EAEC + EPEC	6
*E. coli* O157:H7 + STEC stx1/stx2	7

### Stability study

The stability of stool specimens collected using Para-Pak C&S collection tubes were tested for 5 days at ambient temperature. Forty stool specimens were collected from the same subjects and analyzed before and after the shipment (shipped on April 07, 2015 and received at Vibrant America on April 14, 2015). DNA/RNA from fecal samples collections were extracted before and after shipment. The DNA/RNA from all the extractions were used to run stool culture and RT-PCR based assays (operation procedures in Additional files 1, 2) and compared to ensure there was no impact on the accuracy of the results after shipping and handling process. Concordance between the expected genotypes and that determined after shipping and handling was 100% as shown in Table 8. The detailed stability study results are in the Additional file 3.

**Table 8:** Five-day stability test of stool specimens

Assay	Number of total samples	Number of correct samples	Percent of correct calls
Before shipping			
Culture and RT-PCR	28	28	100
After shipping			
Culture and RT-PCR	28	28	100

Overall, these data demonstrate that this DNA microarray is capable of accurately detecting bacterial, viral, parasitic, and fungal pathogens directly from a stool specimen in enteric transport medium at 96 patient samples per instrument per hour with an additional strength of targeting 27 pathogens simultaneously.

## Discussion

Molecular diagnostics have emerged to play a significant role in detection of infectious diseases. US Food and Drug Administration (FDA) has approved various nucleic acid amplification tests for diagnosis of bacterial, mycobacterial, and viral infections. There has been a particular interest for molecular diagnostics for diarrhea, where higher sensitivity and lower cost is required. Several PCR-based multiplex panels for etiologies of gastroenteritis have been approved by the FDA [12, 13]. The unique advantage of these multiplex PCRs is their ability to detect a wide variety of pathogens in a single panel. The FDA-cleared panels on the current market usually allow for the detection and identification of up to 20 pathogens in 1–5 h turnaround time. The xTAG GPP assay has a test menu of 14 FDA-cleared targets while it has 45 min hands-on time and 5 h turnaround time [14]. A major issue with this assay is that conventional bacterial culture and parasitological examination are still required for several major pathogens [13]. The Verigene EP assay includes only 9 FDA-cleared targets but it is designed to test one sample per processor with 2 h turnaround time. The FilmArray GI panel represents 22 FDA-cleared targets in a closed reaction vessel with results available in 60 min for one patient sample [12], which limits its application in breakouts or other situations requiring high test volumes. Additionally, there were reproducibility and accuracy issues with several species in most currently available multiplex gut pathogen panels [13]. The presented Vibrant GPP uses a semiconductor microarray-based assay and the tests are carried out in a College of American Pathologists (CAP) and Clinical Laboratory Improvement Amendments (CLIA) certified in-house laboratory. This assay is capable to simultaneously process 96 patient samples per instrument per hour with an additional strength of targeting 27 pathogens. The core technology of the Vibrant GPP is the DNA microarray that is easy to incorporate new probes when new pathogens are emerging. This ultra-high-density microarray also provides an unprecedented platform that is universal for all similar applications which are in need of high throughput and low cost.

In this study, we aimed at evaluating the performance of our DNA microarray when compared to conventional methods in clinical laboratories. The manufacturing of the DNA microarray is similar to the fabrication of a peptide microarray described in our previous publications [15, 16] while it employed nucleotide building blocks (A, T, C, G) instead of amino acids. The Vibrant GPP is an expanded GI pathogen panel consisted of multiple species that were not included in any commercially available GI panels as of February 2019. Two bacteria (*Helicobacter pylori*, *Listeria* spp.) and two fungi (*Candida* spp., *Microsporidium* spp.) may provide new information when facing emerging clinical difficulties. The LoDs of the assay range from 10^2^ to 10^4^ cells/mL for bacterial DNA, 10^2^ to 10^3^ cells/mL for parasital DNA, 10^2^ to 10^3^ RNA copies/mL for viral RNA, and 10^2^ to 10^3^ cells/mL for fungal DNA. The LoDs were equal to or tenfold lower than those of comparable commercial gut pathogen panels [13]. The Vibrant GPP was able to detect culture/PCR-confirmed isolates while maintaining a high degree of sensitivity and specificity.

**Fig. 1:**
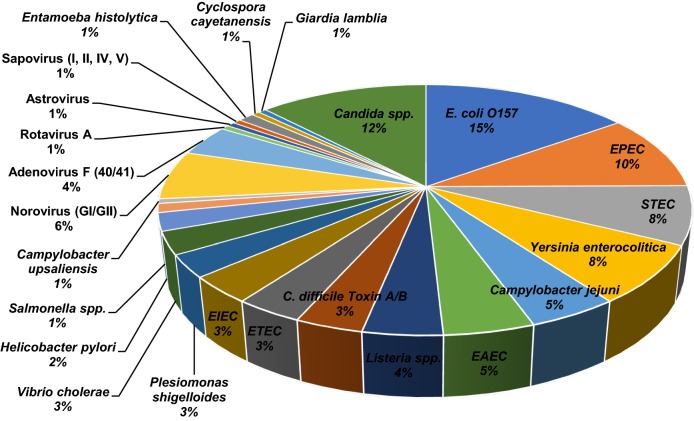
Incidence of pathogens present in clinical stool specimens detected by the Vibrant GPP

We have determined the Vibrant GPP’s analytical performance by testing reproducibility and sensitivity with previously confirmed culture isolates. To further investigate the performance of the panel, the Vibrant GPP was evaluated in terms of capacity to detect diarrhea-related pathogens in stool specimens. A large pool of clinical specimens and archived specimens were confirmed by culturing and RT-PCR methods. The Vibrant GPP detected 23 out of 27 targeted genes (incidences shown in Fig. 1), whereas 4 targets were not detected in the initial pool but verified through the pool of archived specimens. One significant issue of using PCR to detect stool DNAs is that PCR inhibitors such as bile salts and polysaccharides are often present in stool specimens [17]. PCR inhibitors can dramatically reduce the sensitivity and amplification of PCR [18]. The presented assay overcomes this issue through hybridizing the DNA sequences to the high-density sequence-specific probes which could capture the sequences more specifically. Furthermore, an on-chip enzyme-based labelling technique along with the chemiluminescence detection system amplifies the signals of low-leveled sequences and enables improved level of assay sensitivity. The results obtained with all 27 targets in the assay panel were repeatable and reliable.

The introduction of GI PCR panel in the clinical testing algorithms has considerably reduced both the turnaround time and overall economic burdens [19]. The capability of detecting multiple pathogens can be valuable to assist treatment of polymicrobial infections associated with diarrhea, which occur very frequently among young children [20]. The high throughput of the DNA microarray-based Vibrant GPP enables efficient screening of a broad range of diarrhea-related enteric pathogens and provide etiological information for non-diarrhea control samples. Additional pathogen information may improve overall patient care through offering efficient treatment regimens and/or reducing secondary infections and failed treatments.

In conclusion, a strategy with an extensive menu of pathogens that improves sensitivity, limit of detection, turnaround time, and workflow is presented. The Vibrant GPP has been demonstrated to be suitable as a primary detection tool for enteric bacteria, viruses, fungi, and parasites. The sensitivity was shown to be equivalent to or better than conventional methods employed by reference laboratories. With 95.9% sensitivity and 100% specificity, we believe that this GI panel of 27 pathogens has provided an unprecedented opportunity for rapid detection of stool specimens during routine and/or outbreaks investigations. The versatility of this DNA microarray will be useful for streamlining highly reliable, accurate, and actionable detection algorithms of extensive pathogens involved in respiratory, encephalitis/meningitis, pneumonia, and other comparable conditions.

## Supplementary information

**Additional file 1.** Standard operating procedure for stool culture.

**Additional file 2.** Real-time polymerase chain reaction (RT-PCR) operation procedure.

**Additional file 3.** Stability study of stool specimens.

## Data Availability

The data used to support the findings of this study are included within the article.

## Abbreviations

CI: confidence interval
GI: gastrointestinal
GPP: GI pathogen panel
PCR: polymerase chain reaction
ATCC: American Type Culture Collection
HRP: horseradish peroxidase
LoD: limit of detection
*E. coli* O157: *Escherichia coli* O157:H7
EAEC: Enteroaggregative *Escherichia coli*
EPEC: Enteropathogenic *Escherichia coli*
ETEC: Enterotoxigenic *Escherichia coli*
STEC: Shiga-like toxin producing *Escherichia coli*
EIEC: Shigella/Enteroinvasive *Escherichia coli*
