# Supplementary material for: Evaluation of the Vibrant DNA microarray for the high-throughput multiplex detection of enteric pathogens in clinical samples
Source: Gut Pathog. 2019 Oct 18;11:51. doi: 10.1186/s13099-019-0329-2 (PMC6798489; doi:10.1186/s13099-019-0329-2)
Supplement: Supplementary file 1 — Additional file 1. Standard operating procedure for stool culture. [file 13099_2019_329_MOESM1_ESM.docx]

**Additional Information 1**

Standard Operating Procedure for Stool Culture

The purpose of the stool culture is to detect the presence of pathogenic [bacteria](https://labtestsonline.org/glossary/bacterium/), fungi and help diagnose an infection of the digestive system (gastrointestinal, GI tract). This procedure applies to all authorized laboratory personnel authorized to perform the stool culture.

**CONSUMABLES, EQUIPMENT, AND REAGENTS**

Consumables:

| **Consumable** | **Vendor** | **Catalog Number** |
| --- | --- | --- |
| Disposable sterile loops and needles | VWR | 30002-092/098 |
| Microscope slides | VWR | 16005-106 |
| Immersion oil | Amazon | 881314886944 |
| BD GasPak Incubation chamber | VWR | 90003-634 |
| BD GasPak Campy sachets | VWR | 90003-646 |
| BD GasPak Anaerobe sachets | VWR | 90003-642 |
| BD GasPak Anaerobic indicator strips | VWR | 90003-866 |
| McFarland standard kit | Biomerieux | Ref 70900 |

Equipment:

| **Equipment** | **Vendor** | **Catalog Number** |
| --- | --- | --- |
| TSX Series High Performance Lab Refrigerator | Thermo Scientific | 75839-042 |
| Biological safety cabinet | Labconco | 3440009 |
| Incubator 37°C | VWR | 89511-426 |
| Incubator 42°C | VWR | 89511-424 |
| Microscope | OMAX | N/A |
| Slide warmer | Premiere | Model XH-2002 |

Reagents:

| **Reagent** | **Vendor** | **Part/Catalog Number** | **Storage** |
| --- | --- | --- | --- |
| API 20E reagent kit | Biomerieux | Ref 20 120 | 2-8°C |
| E.coli 0157 Latex kit | Thermo fisher | DR0620M | 2-8°C |
| API 20E | Biomerieux | Ref 20 100 | 2-8°C |
| API 20A | Biomerieux | Ref 20 300 | 2-8°C |
| API Campy | Biomerieux | n/a | 2-8°C |
| Gram Stain Kit Advanced by Hardy | VWR | 89407-604 | Room Temp |
| API NaCl 0.85% Medium, 5 ml | Biomerieux | Ref 20 230 | Room Temp |
| Mineral oil | Biomerieux | Ref 70 100 | Room Temp |
| Hydrogen Peroxide 3% | VWR | BDH7540-2 | Room Temp |
| OxiDrops, liquid oxidase reagent | VWR | 89428-918 | 2-8°C |
| Indole, dmaca spot, Hardy | VWR | 89428-932 | 2-8°C |
| Wellcolex Colour Shigella | Thermoscientific | R30858301 | 2-8°C |

Culture Media:

| **Media** | **Vendor** | **Part/Catalog Number** | **Storage** |
| --- | --- | --- | --- |
| Blood Agar Plate 5% Sheep Blood in TSA | Hardy Diagnostics | A10BX | 2-8°C |
| MacConkey Agar | Hardy Diagnostics | G35 | 2-8°C |
| HE (Hektoen Enteric) Agar | Hardy Diagnostics | G63 | 2-8°C |
| MacConkey Agar with Sorbitol | Hardy Diagnostics | G36 | 2-8°C |
| Germ Tube Cryo | Hardy Diagnostics | Z217 | -80°C |
| Emmon’s Sabdex Agar | Hardy Diagnostics | W20 | 2-8°C |
| CCFA (Cycloserine-Cefoxitin Fructose Agar for C.diff. | Hardy Diagnostics | AG501 | 2-8°C |
| Campy CVA Agar with 5% sheep blood | Hardy Diagnostics | A40 | 2-8°C |
| Columbia CNA Agar | Hardy Diagnostics | A50 | 2-8°C |
| Brucella Blood Agar with Hemin and Vitamin K | Hardy Diagnostics | A30 | 2-8°C |
| CIN Agar (Cefsulodin-Irgasan-Novobiosin) | Hardy Diagnostics | G20 | 2-8°C |
| GN Broth, 10ml fill | Hardy Diagnostics | K39 | 2-8°C |
| Urea Agar Slant | Hardy Diagnostics | R42 | 2-8°C |
| Bile Esculin Agar Slant BEA | Hardy Diagnostics | L10 | 2-8°C |
| BBL Motility Test Medium, BD | VWR | 90001-376 | 2-8°C |
| TCBS (thiosulfate citrate bile salts sucrose) agar | Hardy Diagnostics | G55 | 2-8°C |
| CPC-Agar (Base) | sigmaaldrich | 17134 | 2-8°C |

**PROCEDURE**

**Culture and Isolation of stool pathogen:** Appropriate media was equilibrated to room temperature prior to use. Stool samples were inoculated to the appropriate medium (refer to SI Table 1.1) using sterile inoculating loop and spread the material into four quadrants on the plate with a back and forth motion in each quadrant. The plates were incubated on appropriate temperature for 24-48 hours. Anaerobic organisms must be incubated under anaerobic conditions at 37°C for 48 hours. Campylobacter must be incubated at 42°C for 48 hours under microaerophilic conditions. After incubation, the pathogenic bacteria can be differentiated based on colony morphology, fermentation, color on specific media type. The colony in question were isolated, gram stained, and incubated at appropriate temperature and condition.

**Identification Procedure:** Fresh culture (18-24 hours old, or as soon as acceptable growth is seen) was used for bacterial identification. Gram staining was used to differentiate two large groups of bacteria based on their different cell wall constituents.

**Oxidase Test:** One drop of the reagent was added on a piece of filter paper. A good-sized amount of an isolated colony was picked from a plate and spreaded on the filter paper. A positive reaction was indicated by a bluish-purple color which occurred within 30 seconds. No reaction was read after 30 seconds.

**Indole test:** One drop of the reagent was added on a piece of filter paper. A good-sized amount of an isolated colony was picked from a plate and spread it on the filter paper. A positive reaction was indicated by a bright blue greenish color which occurred within 30 seconds. No reaction was read after 30 seconds.

**Catalase Test:** One or two colonies were picked with a sterile loop or needle and spreaded on a microscopic slide. One drop of 3% Hydrogen Peroxide was added. The formation of immediate evolution of gas bubbles was indicative of a positive test.

**Bile Esculin:** Bile salts are the selective ingredient, while [esculin](https://en.wikipedia.org/wiki/Esculin) is the differential component. [*Enterococcus*](https://en.wikipedia.org/wiki/Enterococcus) hydrolyze esculin to products that react with ferric citrate in the medium to produce insoluble iron salts, resulting in the blackening of the medium. Test results were interpreted in conjunction with [gram stain](https://en.wikipedia.org/wiki/Gram_stain) morphology.

**Urea:** Urea is the product of decarboxylation of amino acids. Hydrolysis of urea produces ammonia and CO_2_. The formation of ammonia alkalinized the medium, and the pH shift was detected by the color change of phenol red from light orange at pH 6.8 to magenta (pink) at pH 8.1. Rapid urease-positive organisms turned the entire medium pink within 24 hours.

**Motility:** Bacteria that possess flagella have the ability to move and thus are motile. Live wet mount preparations of a bacterial culture may be viewed under a microscope under oil immersion to determine if an organism is motile. An alternative method, one that safer when working with potential pathogens, is motility stab. A sterile, inoculating needle was used to obtain inoculum from a pure culture of the test organism. The needle was stabbed into motility media approximately two-thirds of the depth of the media. The needle was then pulled out of the media as close as possible to the location where it entered. The tube was incubated for approximately 1-2 days and observed for evidence of motility. A non-motile organism had a clearly defined edge as it grew on the stab line. Motile organisms were turbid throughout the tube or had fuzzy, diffuse growth at the edges.

**API Identification:** API is a standardized identification system consists of 20 microtubes containing dehydrated substrates. These tests were inoculated with a bacterial suspension that reconstitutes the media. During incubation, metabolism produced color changes that were either spontaneous or revealed by the addition of reagents. The reactions were read according to the Reading Table and the identification is obtained by referring to the Analytical Profile Index or using the Identification software.

**SI Table S1.1**. Identification chart for stool pathogen cultures.

| **Stool Pathogen** | **Media to set-up, colony morphology and fermentation/color on media** | **Incubation temperature and condition** | **Gram Stain** | **Supplemental tests** | **Identification** |
| --- | --- | --- | --- | --- | --- |
| Shigella sp. | BAP: gray  MAC: Non-fermenter/no color  HE: non-fermenter/no color  GN Broth: turbid | All at 37 °C aerobically | Gram Negative Rod | Indole: Negative Oxidase: Negative | API 20E,  Wellcolex Color Shigella |
| Helicobacter pylori | BAP: no growth or pin point. Brucella: small, moist | BAP-37°C aerobically Brucella -37°C microaerophilic | Curved Gram Negative Rod | Oxidase: Positive Catalase: Positive Urea: Positive | API Campy |
| Enterotoxigenic E. coli | BAP: gray MAC: fermenter/pink  SMAC: fermenter/pink | All at 37 °C aerobically | Gram Negative Rod | Indole: Positive Oxidase: Negative | API 20 E |
| Clostridium difficle | BAP aer.: no growth  CCFA: yellow colonies/horse manure smell  BAP ana.: flat gray | BAP aer 37°C aerobically  CCFA and BAP ana: 37°C anaerobically | Gram Positive Rods with spores | Catalase: Negative | API 20A |
| Campylobacter sp. | BAP aer.: no growth  CVA: moist colonies along the streak  BAP ana.: moist, watery colonies | BAP aer 37°C aerobically  CVA and BAP ana: 42°C microaerophilic | Curved Gram Negative Rod | Oxidase: Positive Catalase: Positive | API Campy |
| E. coli O157 | BAP: gray MAC: fermenter/pink  SMAC: non-fermenter/no color | All at 37 °C aerobically | Gram Negative Rod | Indole: Positive Oxidase: Negative | API 20E,  E. coli O157 Latex |
| Yersinia enterocolitica | BAP: small gray  CIN: red centered small | BAP-37°C aerobically  CIN: 25°C room temp. | Gram Negative Rod | Indole: Negative Oxidase: Negative | API 20E |
| Salmonella sp. | BAP: gray  MAC: Non-fermenter/no color  HE: black centered colonies  GN Broth: turbid | All at 37 °C aerobically | Gram Negative Rod | Indole: Negative Oxidase: Negative | API 20E |
| Listeria sp. | BAP: small gray, beta  CNA: small gray, beta | All at 37 °C aerobically | Small Gram Positive Rods | Catalase: Positive Bile Esculin: Positive Motility: Positive at 25°C, Negative or weak at 37°C | N/A |
| Vibrio parahaemolyticus | TCBS agar: Yellow  mCPC agar: no growth | All at 37 °C aerobically | Curved Gram Negative Rod | Oxidase: Positive | N/A |
| Vibrio vulnificus | TCBS agar: Green  mCPC agar: Yellow | All at 37 °C aerobically | Curved Gram Negative Rod | Oxidase: Positive | N/A |
| Vibrio Cholerae | TCBS agar: Yellow  mCPC agar: Purple | All at 37 °C aerobically | Curved Gram Negative Rod | Oxidase: Positive | N/A |

Note:

- Stools for the C. difficle samples must be transported to the laboratory immediately or refrigerated if transport is delayed.
- Only loose or diarrheal stools are recommended for routine bacterial and C. difficle cultures.

**REFERENCES**

- Product Manual: API 20E, API 20A and API Campy
- CLSI guideline: Molecular Diagnostics Methods for Infectious Diseases, MM03-A2
