# Supplementary material for: Evaluation of the Vibrant DNA microarray for the high-throughput multiplex detection of enteric pathogens in clinical samples
Source: Gut Pathog. 2019 Oct 18;11:51. doi: 10.1186/s13099-019-0329-2 (PMC6798489; doi:10.1186/s13099-019-0329-2)
Supplement: Supplementary file 2 — Additional file 2. Real-time polymerase chain reaction (RT-PCR) operation procedure. [file 13099_2019_329_MOESM2_ESM.docx]

**Additional Information 2**

Real-Time Polymerase Chain Reaction (RT-PCR) Operation Procedure

The purpose of this section will provide information necessary to conduct real time-PCR (RT-PCR) in assessing the presence/ absence of the pathogenic Bacteria, virus, parasite and fungi that symbiotically live in the human gastrointestinal tracts.

**Extraction of DNA/RNA from Fecal/ Culture samples**

In this study we used Mag-Bind® Universal Pathogen 96 Kit from Omega Biotek, Norcross, GA for the extraction and purification of total pathogenic DNA/RNA from fecal and culture samples.

**Design of species-specific TaqMan assays**

Primers and probes were designed for all species using the Primer Express 3.0 software for Real-Time PCR (Applied Biosystems Foster City, CA, USA) by using MGB TaqMan probes with unique regions having amplicon length 60 to 120 bp and using the default parameters of the software. The Primer and Probes were purchased from Integrated DNA Technologies, Inc and Custom TaqMan Probes from Thermofisher, USA.

**Real-Time PCR amplification of DNA with primers and labelled probes**

Reagents were completely thawed at room temperature. The amplification mix tube was vortexed for 2 to 5 seconds then centrifuged briefly to bring the contents to the bottom of the tube. The PCR master mix was prepared with 8 µL TaqMan Genotyping Master Mix (PN 4371131B) and 1 µL Probe Mix Primer. This 9 µL PCR master mix was then gently vortexed and dispensed into each well of the PCR well plate. Then 1 µL DNA template was added to each well. This mixing process was repeated for each individual well. Once all wells have been prepared, the well plate was placed in a tray and sealed in a quant studio 5 RTPCR system. Immediately the amplification reaction was commenced using the following program.

| **Step No.** | **Temperature °C** | **Time** | **No. of cycles** |
| --- | --- | --- | --- |
| 1 | 95 | 3 min. | N/A |
| 2 | 95  55  72 | 30 sec.  30 sec.  30 sec. | 40x |
| 3 | 72 | 7 min. | 1x |
| 4 | 4 | 120 sec. | 1x |

**Real-Time PCR amplification of RNA with primers and labelled probes**

All reagents were completely thaw at room temperature. The amplification mix tube was vortexed for 2 to 5 seconds then centrifuged briefly to bring the contents to the bottom of the tube. The PCR master mix for each primer mix was prepared with 5 µL TaqMan RT-PCR Mix (2x), 0.25 µL TaqMan RT Enzyme Mix (40x), 1 µL Primer Probe Mix, 2 µL RNA template, and 1.75 µL RNase-free H_2_O. This mixing process (PN 4393463D) was repeated for each individual well. Once all wells have been prepared, the well plate was placed in a tray and sealed in a quant studio 5RTPCR system. Immediately the amplification reaction was commenced using the following program.

| **Step No.** | **Temperature °C** | **Time** | **No. of cycles** |
| --- | --- | --- | --- |
|  | 48 | 15min. | N/A |
| 1 | 95 | 10min. | N/A |
| 2 | 95  55  72 | 15 sec.  30 sec.  30 sec | 40x |
| 3 | 72 | 7 min. | 1x |
| 4 | 4 | 120 sec. | 1x |

**Result interpretation**

The presence of the target species was determined for RT-PCR with Ct values <35. With Ct values between 34 and 35 the species was considered to be present at LOD levels, and values of Ct>35, which is the cut off limit, associated with no detection.
