# Supplementary material for: Evaluation of the Vibrant DNA microarray for the high-throughput multiplex detection of enteric pathogens in clinical samples
Source: Gut Pathog. 2019 Oct 18;11:51. doi: 10.1186/s13099-019-0329-2 (PMC6798489; doi:10.1186/s13099-019-0329-2)
Supplement: Supplementary file 3 — Additional file 3. Stability study of stool specimens. [file 13099_2019_329_MOESM3_ESM.docx]

**Additional Information 3**

Stability Study of Stool Specimens

The purpose of this study is to check the stability of stool specimens collected using Para-Pak C&S collection tubes for 5 days at ambient temperature. DNA/RNA from fecal samples collections were extracted from the same patients before and after shipment. The DNA /RNA from all the extractions were used to run stool culture and RT-PCR based assays and compared to ensure there was no impact on the accuracy of the results for 5-day storage/shipment period.

**PROCEDURE**

Collect fecal samples from the same patients as below for analysis before and after shipping of the specimens. After extraction process, all samples were run with an assay to determine if the accuracy of the procedure is more than 90%. Accuracy for this protocol is defined as the closeness of agreement of results between the fecal samples before and after shipment. The samples were shipped on 4/07/2015 and received on 4/14/2015.

**RESULTS**

The tables in this section detail the results of the stool specimen validation runs before and after the shipping and handling.

**SI Table S2.1.** Fecal samples – DNA Concentration Before and After shipment.

| **Sample ID** | **Pathogenic Bacteria** | **Concentration Before Shipment (ng)** | **Concentration After Shipment (ng)** |
| --- | --- | --- | --- |
| Pathogen1001 | Clostridium difficile toxin A | 9.23 | 4.36 |
| Pathogen1002 | Clostridium difficile toxin B | 12.36 | 3.53 |
| Pathogen1003 | Campylobacter spp. (jejuni, coli, and upsaliensis) | 2.5 | 3.75 |
| Pathogen1004 | Plesiomonas shigelloides | 6.25 | 5.01 |
| Pathogen1005 | Yersinia enterocolitica | 10.23 | 7.5 |
| Pathogen1006 | Salmonella spp. | 6.25 | 3.75 |
| Pathogen1007 | Vibrio parahaemolyticus | 3.75 | 8.75 |
| Pathogen1008 | Vibrio cholerae | 8.75 | 6.25 |
| Pathogen1009 | Vibrio vulnificus | 6.25 | 3.75 |
| Pathogen1010 | Enteroaggregative E. Coli (EAEC) | 8.75 | 5.01 |
| Pathogen1011 | Enteropathogenic E. Coli (EPEC) | 8.11 | 4.25 |
| Pathogen1012 | Enterotoxigenic E. Coli (ETEC) lt/st | 7.5 | 4.65 |
| Pathogen1013 | Shiga-like toxin producing E. Coli (STEC) | 3.11 | 2.36 |
| Pathogen1014 | E. Coli O157 | 2.5 | 4.65 |
| Pathogen1015 | Shigella/Enteroinvasive E. Coli (EIEC) | 8.75 | 8.36 |
| Pathogen1016 | Helicobacter pylori | 5.25 | 4.65 |
| Pathogen1017 | Listeria spp. | 6.25 | 8.75 |

| **Sample ID** | **Parasite** | **Concentration Before Shipment (ng)** | **Concentration After Shipment (ng)** |
| --- | --- | --- | --- |
| Pathogen1018 | Cryptosporidium | 4.12 | 3.25 |
| Pathogen1019 | Entamoeba histolytica | 14.42 | 2.64 |
| Pathogen1020 | Giardia lamblia | 8.24 | 3.15 |
| Pathogen1021 | Cyclospora cayetanensis | 12.36 | 10.23 |

| **Sample ID** | **Virus** | **Concentration Before Shipment (ng)** | **Concentration After Shipment (ng)** |
| --- | --- | --- | --- |
| Pathogen1023 | Norovirus GI/GII | 1.25 | 2.11 |
| Pathogen1024 | Rotavirus A | 6.4 | 2.35 |
| Pathogen1025 | Adenovirus F 40/41 | 2.05 | 1.36 |
| Pathogen1026 | Astrovirus | 8.45 | 5.22 |
| Pathogen1027 | Sapovirus (I, II, IV, V) | 8.2 | 4.12 |

| **Sample ID** | **Fungi** | **Concentration Before Shipment (ng)** | **Concentration After Shipment (ng)** |
| --- | --- | --- | --- |
| Pathogen1028 | Candida spp. | 4.21 | 3.45 |
| Pathogen1029 | Microsporidium spp. | 10.64 | 9.78 |

**SI Table S2.2.** Gut Pathogen Assay Result summary: DNA Stability Before and After shipment Sample Comparison**.**

| **Sample ID** | **Pathogen (Bacteria/Parasite/Virus/Fungi)** | **Gut Pathogen Assay Result Before Shipment** | **Gut Pathogen Assay Result After Shipment** |
| --- | --- | --- | --- |
| Pathogen1001 | Clostridium difficile toxin A | Detected | Detected |
| Pathogen1002 | Clostridium difficile toxin B | Detected | Detected |
| Pathogen1003 | Campylobacter spp. (jejuni, coli, and upsaliensis) | Detected | Detected |
| Pathogen1004 | Plesiomonas shigelloides | Detected | Detected |
| Pathogen1005 | Yersinia enterocolitica | Detected | Detected |
| Pathogen1006 | Salmonella spp. | Detected | Detected |
| Pathogen1007 | Vibrio parahaemolyticus | Detected | Detected |
| Pathogen1008 | Vibrio cholerae | Detected | Detected |
| Pathogen1009 | Vibrio vulnificus | Detected | Detected |
| Pathogen1010 | Enteroaggregative E. Coli (EAEC) | Detected | Detected |
| Pathogen1011 | Enteropathogenic E. Coli (EPEC) | Detected | Detected |
| Pathogen1012 | Enterotoxigenic E. Coli (ETEC) lt/st | Detected | Detected |
| Pathogen1013 | Shiga-like toxin producing E. Coli (STEC) | Detected | Detected |
| Pathogen1014 | E. Coli O157 | Detected | Detected |
| Pathogen1015 | Shigella/Enteroinvasive E. Coli (EIEC) | Detected | Detected |
| Pathogen1016 | Helicobacter pylori | Detected | Detected |
| Pathogen1017 | Listeria spp. | Detected | Detected |
| Pathogen1019 | Cryptosporidium | Detected | Detected |
| Pathogen1020 | Entamoeba histolytica | Detected | Detected |
| Pathogen1021 | Giardia lamblia | Detected | Detected |
| Pathogen1022 | Cyclospora cayetanensis | Detected | Detected |
| Pathogen1023 | Norovirus GI/GII | Detected | Detected |
| Pathogen1024 | Rotavirus A | Detected | Detected |
| Pathogen1025 | Adenovirus F 40/41 | Detected | Detected |
| Pathogen1026 | Astrovirus | Detected | Detected |
| Pathogen1027 | Sapovirus (I, II, IV, V) | Detected | Detected |
| Pathogen1028 | Candida spp. | Detected | Detected |
| Pathogen1029 | Microsporidium spp. | Detected | Detected |

**CONCLUSION**

All validation assay test results met their required specifications of >90% Accuracy. Therefore, it is acceptable to ship fecal samples within 5 days at ambient temperature before DNA extraction process.
